# Fucoidan Inhibition of Osteosarcoma Cells is Species and Molecular Weight Dependent

**DOI:** 10.3390/md18020104

**Published:** 2020-02-08

**Authors:** Dhanak Gupta, Melissa Silva, Karolina Radziun, Diana C. Martinez, Christopher J. Hill, Julie Marshall, Vanessa Hearnden, Miguel A. Puertas-Mejia, Gwendolen C. Reilly

**Affiliations:** 1 Department of Materials Science and Engineering, University of Sheffield, Sheffield S1 3JD, UK; dhanakgupta@gmail.com (D.G.); radziun.k@gmail.com (K.R.); dinama2012@gmail.com (D.C.M.); v.hearnden@sheffield.ac.uk (V.H.); 2 INSIGNEO Institute for in Silico Medicine, University of Sheffield, Sheffield S1 3JD, UK; julie.marshall@sheffield.ac.uk; 3 Institute of Chemistry, University of Antioquia, Medellín A.A.1226, Colombia; melissa.silva1@udea.edu.co (M.S.); miguel.puertas@udea.edu.co (M.A.P.-M.); 4 Cell Bank, Department of Cell Biology, Faculty of Biochemistry, Biophysics and Biotechnology, Jagiellonian University, 30-387 Krakow, Poland; 5 Department of Molecular Biology and Biotechnology (MBB), University of Sheffield, Sheffield S10 2TN, UK; c.j.hill@sheffield.ac.uk

**Keywords:** apoptosis, necrosis, brown algae, mitochondria, MG63 cells, fucoidan, transmission electron microscopy, molecular weight fraction, crude extract, cell cycle

## Abstract

Fucoidan is a brown algae-derived polysaccharide having several biomedical applications. This study simultaneously compares the anti-cancer activities of crude fucoidans from *Fucus vesiculosus* and *Sargassum filipendula*, and effects of low (LMW, 10–50 kDa), medium (MMW, 50–100 kDa) and high (HMW, >100 kDa) molecular weight fractions of *S. filipendula* fucoidan against osteosarcoma cells. Glucose, fucose and acid levels were lower and sulphation was higher in *F. vesiculosus* crude fucoidan compared to *S. filipendula* crude fucoidan. MMW had the highest levels of sugars, acids and sulphation among molecular weight fractions. There was a dose-dependent drop in focal adhesion formation and proliferation of cells for all fucoidan-types, but *F. vesiculosus* fucoidan and HMW had the strongest effects. G1-phase arrest was induced by *F. vesiculosus* fucoidan, MMW and HMW, however *F. vesiculosus* fucoidan treatment also caused accumulation in the sub-G1-phase. Mitochondrial damage occurred for all fucoidan-types, however *F. vesiculosus* fucoidan led to mitochondrial fragmentation. Annexin V/PI, TUNEL and cytochrome c staining confirmed stress-induced apoptosis-like cell death for *F. vesiculosus* fucoidan and features of stress-induced necrosis-like cell death for *S. filipendula* fucoidans. There was also variation in penetrability of different fucoidans inside the cell. These differences in anti-cancer activity of fucoidans are applicable for osteosarcoma treatment.

## 1. Introduction

Fucoidan is a class of sulphated and fucose-rich polysaccharides usually derived from the fibrillar cell walls and intracellular spaces of brown algae. First isolated by Kylin in 1913 [1], fucoidan has been demonstrated to have several biological properties ranging from anti-cancer, anticoagulant, and antithrombotic, antiviral, immunomodulatory and antioxidant [2]. Several factors can affect the structure of fucoidan, such as the species of seaweed from which it is extracted [2,3], habitat, season of harvest [4] and extraction method [5]. These can alter fucoidan’s molecular weight, monosaccharide composition, position of sulphate ester group, branching pattern and level of sulphation, ultimately affecting its bioactivity [6]. 

Fucoidan from *Fucus vesiculosus* has been the most widely studied. Other than this, fucoidans from *Cladosiphon novae-caledoniae* [7], *Cladosiphon okamuranus* [8], *Sargassum filipendula* [9], *Undaria pinnatifida* [10], *Fucus evanescens* [11], *Sargassum polycystum* [12], *Saccharina japonica* [13], *Himantothallus grandifolius* [14] and *Sargassum muticum* [15] (reviewed in References [16,17,18,19]) have also been investigated. However, fucoidan from *Sargassum filipendula* has not previously been investigated or exploited for biomedical applications. For the first time, this study explores fucoidan from *S. filipendula*, growing abundantly across the coastal regions of Colombia, for its effects on cancer cells. Identification of a medical benefit for this invasive species may have economic and environmental benefits for the region.

Molecular weight fractions of fucoidans have also been shown to affect the bioactivity of fucoidan. Cho et al. [20] compared native forms of two molecular weight (MW) fractions of fucoidan extracts in the ranges of 5–30 kDa and >30 kDa from *U. pinnatifida*, and found that there was almost two times stronger dose-dependent (for doses 200 to 800 µg/mL) antiproliferative activity of 5–30 kDa MW compared to the >30 kDa fraction, against the stomach cancer cell line AGS. Álvarez-viñas et al. [15] recently compared <5, 5–10, 10–30, 30–50, 50–100 and >100 kDa of fucoidan extracts from *S. muticum* for their anti-cancer activity against cervical cancer (HeLa 229), ovarian cancer (A2780), hepatocarcinoma (HepG2) and kidney (LLC-PK1) cells. The results showed that overall, HeLa 229 and A2780 cells were more strongly inhibited than HepG2 and LLC-PK1 cells, and even though the maximal sulphate content was found in the >100 kDa fraction, the cytotoxic activity was maximal for the 5–30 kDa fraction.

Anti-tumor or anti-cancer activities of different fucoidans havebeen widely investigated for breast cancer [7,21,22,23,24], colon/colorectal cancer [3,25,26,27,28], liver cancer [13,29], lymphoma [8] and lung cancer [11,30]. However, the effect of fucoidan on osteosarcoma has only recently been investigated [31,32,33].

Osteosarcoma is the most common type of bone cancer occurring in the proximal humerus (usually around the knee), distal femur and proximal tibia of younger children (more than 5 years of age), teenagers (it is the third most common cancer in adolescence) and older adults [34]. Currently, neoadjuvant and adjuvant chemotherapies, before and after surgery, are used for treating osteosarcoma. Up to 30% of patients with high-grade osteosarcoma may develop local or distal recurrence after therapy and this cancer may metastasise to the lungs. The prognosis is poor with deteriorating quality of life. The 5-year survival rate for osteosarcoma is 60%–70%. Recently, it has been highlighted that cell proliferation, apoptosis, adhesion, invasion and metastasis represent potential biological targets for treating osteosarcoma [35], and that one of the most potent natural drugs for this application may be fucoidan. Fucoidanhas been shown to inhibit cancer cells by inducing apoptosis via several mechanisms [21,22,24,29,33,36,37,38,39,40,41], such as inhibition of cell proliferation and invasion by targeting cell cycle proteins and the PI3K-Akt-mTOR pathway [42], inhibition of Vascular Endothelial Growth Factor and Matrix Metalloproteinases [30], enhancing ubiquitin-dependent TGFβ receptor degradation [23,43] and regulating the miR-29c/ADAM12 and miR-17-5p/PTEN axes [44].

This study simultaneously compares the anti-cancer activities of crude fucoidans from two brown seaweed species *F. vesiculosus* and *S. filipendula*, and the effects of low-, medium- and high-MW (LMW, MMW and HMW) fractions of fucoidan derived from *S. filipendula* against MG63 osteosarcoma cells. Using size-fractionated fucoidan, we also demonstrate that as the molecular weight of fucoidan increases, the anti-cancer activity of fucoidan also increases.

## 2. Results

### 2.1. Natural Sugars, Sulphation and Acid Contents in Fucoidan

Comparison of neutral sugars, sulphation and acid contents in different fucoidans indicated that the lowest levels of neutral sugars and acid were present with the highest levels of sulphation (1.5 to 2 times more) in the case of crude extract from *F. vesiculosus* compared to all other fucoidans (Table 1).

### 2.2. Effect of Fucoidan on MG63 Cell Attachment and Morphology

**Figure 1:**
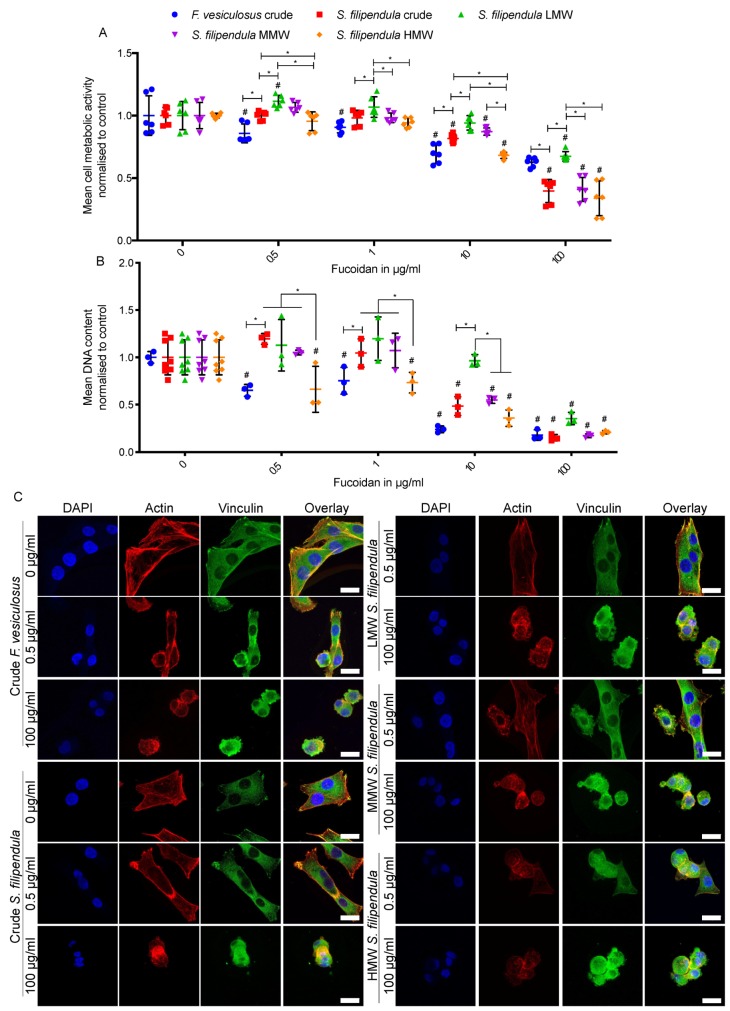
Effect of different fucoidans on MG63 cell attachment. Mean ± SD of (**A**) metabolic activity and (**B**) DNA content after 24 h of seeding in the presence of fucoidan (*n* = 6). (**C**) Max intensity z-projections for DAPI (blue)-, Actin (Texas Red)- and Vinculin (FITC)-stained cells with overlays, after 24 h of seeding in presence of fucoidan. Scale bars, 25 µm. * *p* < 0.05, # *p* < 0.05 relative to respective vehicle controls.

To assess attachment of MG63 cells in medium supplemented with fucoidans, cells were seeded in media containing a range of doses of different fucoidan types. The results (Figure 1A,B) showed a dose-dependent drop in MG63 metabolic activity and DNA content for all fucoidan types with differences in severity based on fucoidan type. Comparison of the two crude extracts showed a more severe reduction in the case of *F. vesiculosus* for doses up to 10 μg/mL. At a higher dose of 100 μg/mL, there seemed to be similar DNA content in both conditions but more cell metabolic activity in MG63s seeded in the presence of fucoidan from *F. vesiculosus*, which suggested that mitochondria in the latter cells were more active.

Comparison of different molecular weight fractions of fucoidan from *S. filipendula* showed that higher molecular weight led to lower cell attachment, as measured by metabolic activity or DNA content. These trends were visible up to the 10 μg/mL dose, and by 100 μg/mL, cells treated with MMW or HMW had similar cell numbers which were significantly lower than LMW-treated cells.

Immunostaining for actin and vinculin (Figure 1C) showed that in control conditions, MG63 cells tended to have a flat and angular morphology with vinculin being highly expressed at the pointed edges of the cells. In the presence of 0.5 μg/mL of fucoidan from *F. vesiculosus*, MG63 cells appeared to be either more rounded or more elongated due to the accumulation of actin in the cell cortex and vinculin staining was lost. On the other hand, in the presence of 0.5 μg/mL of crude fucoidan from *S. filipendula*, the cells seemed to retain their typical morphology and vinculin expression. When 0.5 μg/mL LMW, MMW and HMW fractions were compared, only MMW and HMW fractions from *S. filipendula* caused irregular and rounded cells. Finally, at a dose of 100 μg/mL, MG63 cells always exhibited a rounded morphology with no typical vinculin expression, irrespective of the type of fucoidan supplemented.

We further calculated the percentage of cells that rounded after 24 h of seeding in the presence of different types of fucoidan. The results (Supplementary Figure S1) suggested that at a 0.5 μg/mL dose, there was a similar percentage of rounded cells in the case of both crude fucoidans. However, a comparison of LMW, MMW and HMW fractions from *S. filipendula* showed that as the molecular weight fraction increased, the percentage of rounded cells also increased. Altogether, these results suggest that the effect of fucoidan on MG63 attachment is dependent upon the species it is derived from and the molecular fraction of fucoidan used.

### 2.3. Effect of Fucoidan on MG63 Cell Growth and Mitochondrial Activity

In a preliminary experiment, cells were seeded and the next day, treatment with different doses of crude fucoidan from *F. vesiculosus* was started (Supplementary Figure S2). After 24 h of exposure, DNA content and cell metabolic activity measurements were performed. The results showed that after only one day, the cells were unable to survive at doses higher than 100 µg/mL. The cells were noted to round up and fragment into smaller debris. Though the DNA measurements showed a lower mean value for 100 µg/mL compared to control, this difference was not statistically significant, and some cell debris was also visible at this dose. Hence, for further investigations, concentrations were restricted to 100 µg/mL of fucoidan or less.

**Figure 2:**
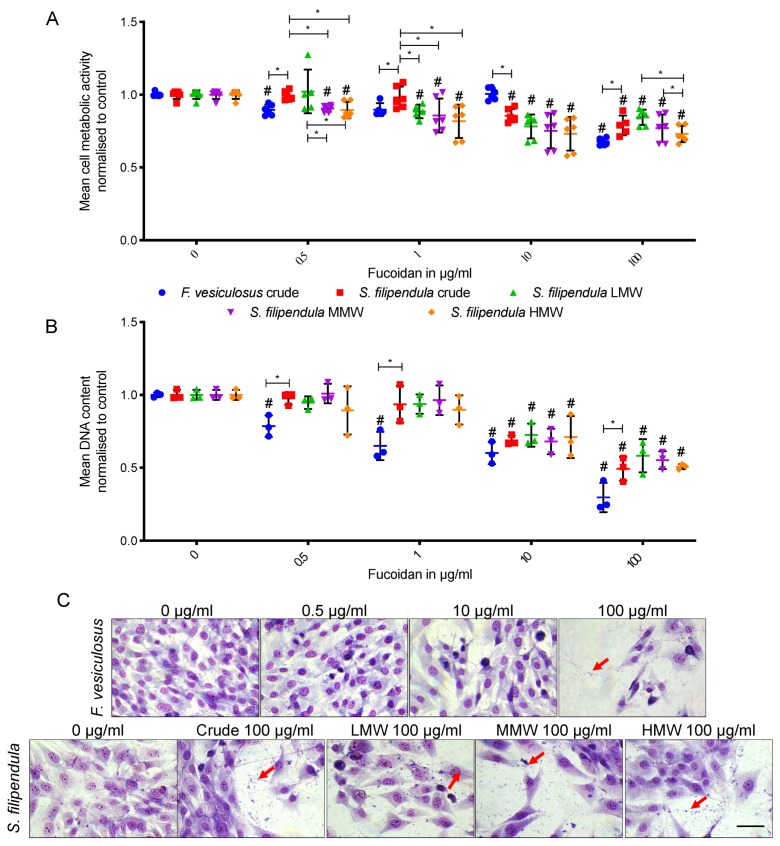
Mean ± SD of (**A**) cell metabolic activity (*n* = 6) and (**B**) DNA content (*n* = 3) of MG63 cells after 3 days treatment with different fucoidans, where fucoidans were applied on day 1. (**C**) Giemsa-stained MG63 cells after 3 days treatment with different fucoidans. Scale bar—50 μm. * *p* < 0.05, # *p* < 0.05 relative to respective vehicle controls. Red arrows—cell debris.

For comparison of different types of fucoidan, experiments were extended up to three days of culture and cells were grown in media supplemented with 0 to 100 μg/mL of different fucoidans. Cell metabolic activity measurements (Figure 2A) also showed a dose-dependent effect on metabolic activity after treatment with all types of fucoidan, with the lowest activity relative to controls for cells treated with 100 μg/mL of crude fucoidan from *F. vesiculosus*. Additionally, there seemed to be a molecular weight-dependent effect of fucoidan from *S. filipendula* on cell metabolic activity. At 1, 10 and 100 μg/mL, there was higher metabolic activity for cells treated with crude and/or LMW fucoidan extracts, followed by the MMW fraction and metabolic activity was lowest in the case of cells treated with the HMW fraction.

DNA content measurement confirmed the metabolic activity data (Figure 2B). There was a dose-dependent effect on cell numbers after treatment with all types of fucoidan; however, the strongest effect was seen in the case of crude fucoidan from *F. vesiculosus*. Additionally, by 100 µg/mL, there was no significant difference between cells under any of the *S. filipendula* fucoidans treatment. 

Giemsa staining was performed to study the cell morphology after three days of exposure to fucoidan. Cells in control conditions had clearly defined nuclei with flat morphology, indicative of good cell health (Figure 2C), whereas cells exposed to 0.5, 10 and 100 µg/mL of crude fucoidan from *F. vesiculosus* had some cells with dark and condensed nuclei with debris surrounding them, suggestive of cell death. In the case of different fucoidan from *S. filipendula*, a dose-dependent effect was seen, and at 100 µg/mL, there was evidence of cell death.

### 2.4. Ultrastructure Examination of MG63 Cells Treated with Fucoidan

**Figure 3:**
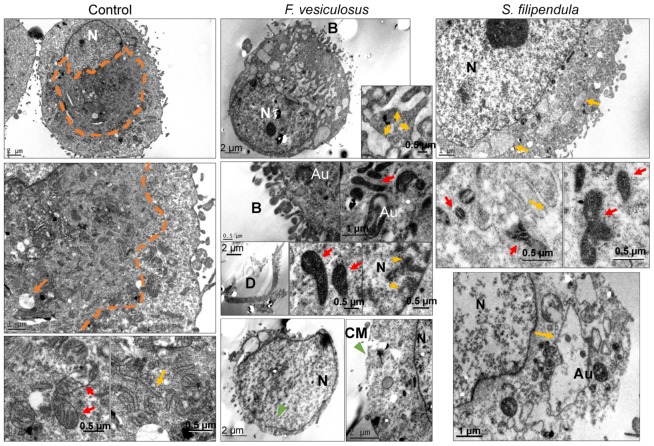
TEM of MG63 cells treated with 100 μg/mL of crude fucoidan from *F. vesiculosus* or *S. filipendula* (at least 10–15 cells analysed per condition). Red arrows—mitochondria, orange arrows—vesicles or vacuoles, orange dashed line—perinuclear region rich in organelles in control cells, N—nucleus, B—blebbing in the cell membrane, D—cellular debris, yellow arrow heads—chromatin condensation and marginalisation, green arrow heads—membrane nicks, yellow arrows—endoplasmic reticulum at higher magnification.

To further understand the effect of fucoidan on cell ultrastructure and organelle organisation, TEM was performed for cells treated with fucoidan for three days (Figure 3). In control (0 μg/mL) MG63 cells, the cytosol near the nucleus was grainy and seemed to consist of cell organelles, which were close to the nucleus rather than dispersed through the cytosol. Higher magnification electron micrographs showed that mitochondria in normal cells had regular structure with clear cristae formation and an extensive endoplasmic reticulum (ER) network in the cytosol. The maximum size of vesicles in the cytoplasm was observed to be around 1 μm by visual inspection. For MG63 cells treated with 100 μg/mL of crude fucoidan from *F. vesiculosus*, cells that were intact had visible signs of blebbing in the cell membrane, extensive ER-like swelling, signs of autophagosome formation, chromatin condensation and marginalisation. Some other cells had broken cell or nuclear membranes and their cytoplasm was less granular or dense. The granularity of the nucleoplasm also became less in cells that appeared to be dying. The mitochondria in all these cells were swollen, very dense and seemed to be disintegrating and losing their cristae structure, which appeared like a dense matrix within the cytosol. Moreover, their distribution was visibly less in the cytosol as compared to mitochondria in the control. There were also signs of cell debris and fragments, possibly of these ruptured cells, within the same sample. When these cells were compared to the cells treated with crude fucoidan from *S. filipendula*, again there seemed to be signs of significant cell death but with some differences. The occurrence of blebs in the cells was less common and there were more cells in the sample with lost cell membrane integrity or rupturing. These cells, as well as some other less damaged cells, seem to have visible ER-like swelling and less dense cytoplasm. The mitochondria were also very dense and swollen and their cristae structure seemed to be collapsing into a dense matrix within the mitochondria. However, other mitochondria had a visible cristae structure that was not fully disintegrated but lacked orientation and were marginalised towards the edges of the mitochondrial membrane, leading to development of gaps inside the mitochondria. The nucleoplasm was less granular and there were also regions of condensed filaments, which may be cytoskeletal actin-filaments in the cytosol. There were also signs of autophagosomes in a few cells, although this was not common. These results indicated that while both crude fucoidans at 100 μg/mL were leading to cell death, their mechanism of action may be different. 

**Figure 4:**
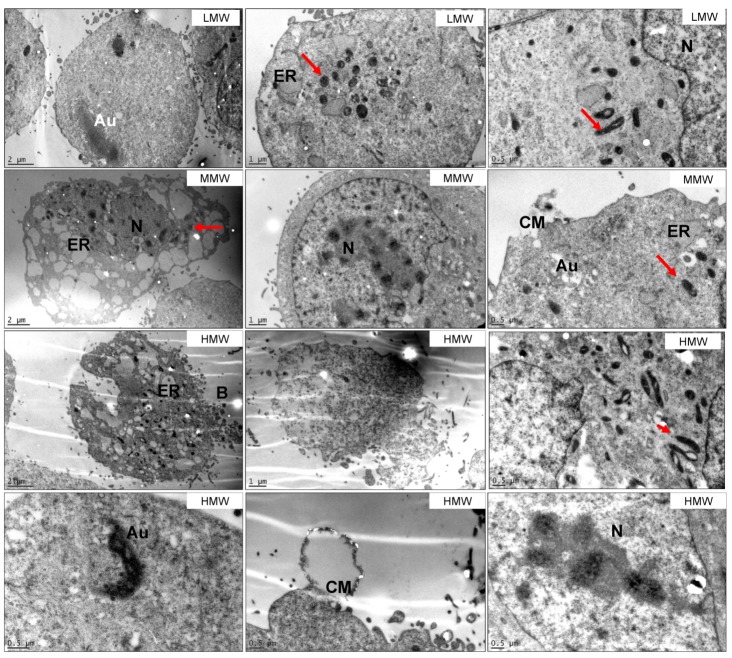
TEM of MG63 cells treated with 100 μg/mL of LMW, MMW and HMW fractions of fucoidan from *S. filipendula* (at least 10 cells were analysed per condition). Different features of structural damage are indicated. Red arrows—mitochondria, N—nucleus, A—condensed actin filaments, ER—endoplasmic reticulum, Au—autophagosomes, B—blebbing, CM—damaged cell membrane.

TEM was also carried out to study the effects of 100 μg/mL of LMW, MMW and HMW fucoidans from *S. filipendula* on MG63 cells (Figure 4). For all three fucoidan types, there were a number of cells with condensation of the actin-like filaments in the cytosol. However, it was seen more for MMW- and HMW-treated samples. The mitochondria were again dense and seemed to have swollen cristae structures, which were disintegrating in more damaged cells. There was also the presence of autophagocytosis in a few treated cells, characterised by highly dense granulated structures or vesicles containing cellular material in the cytoplasm. Moreover, swelling in the cells with ruptured cell membranes and leaking cytoplasm were also a common feature for all fucoidan types. One of the typical features of the intact or dying cells treated with MMW or HMW was the presence of dark spots in the chromatin material in the nucleus, suggestive of fragmented nucleolus or upregulated ribosomal biosynthesis. There were also some cells with blebs in the MMW- and HMW-treated samples, however, this was not a common feature. Altogether, these results confirm that the MMW and HMW fractions of the crude fucoidan from *S. filipendula* were more toxic than the LMW fraction.

### 2.5. Effect of Fucoidan on Cell Cycle

**Figure 5:**
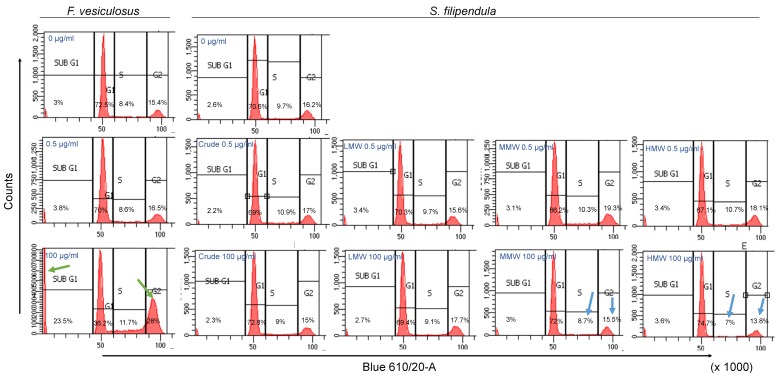
Cell cycle analysis of MG63 cells after 3 days treatment with different fucoidans at 0, 0.5 and 100 μg/mL. Nearly 10,000 single cell events were measured after staining with PI (*n* = 1). Populations were split into sub-G1, G1, S and G2 phases. Notice the presence of more cells in sub G1, S and G2 phases (green arrows) and fewer cells in G1 phase in after treatment with 100 μg/mL of crude fucoidan from *F. vesiculosus* relative to other conditions. There were also fewer cells in S and G2 phases and more cells in G1 phase after treatment with 100 μg/mL of MMW or HMW (blue arrows) derived from *S. filipendula* compared to untreated condition or lower doses of same fucoidans.

PI staining was performed to investigate the effect of fucoidan on the MG63 cell cycle. After three days of treatment with crude fucoidan from *F. vesiculosus*, there were nearly 70%–72.5%, 8.4%–8.6%, 16.5%–15.4% and 3%–3.8% cells in the G1, S, G2 and sub-G1 phases respectively, for doses up to 0.5 μg/mL (Figure 5). However, at 100 μg/mL, these values changed to 36.2%, 11.7%, 28% and 23.5%, respectively. The dramatic changes in population distribution indicated cell cycle arrest in G1 phase that inhibited the cells from entering the S phase and accumulation of cells in the sub-G1 population. In contrast, treatment with 100 μg/mL of crude or molecular weight fractions of fucoidan from *S. filipendula* showed no significant variation in sub-G1 phase compared to lower doses. However, there seemed to be fewer cells present in S and G2 phase and more cells in G1 phase at 100 μg/mL of MMW and HMW fractions compared to lower concentrations, again indicative of arrested cells in G1 phase of cell cycle [45]. A confirmatory cell cycle analysis on Day 2 of treatment with *S. filipendula* fucoidans was also performed to validate these results (Supplementary Figure S3).

### 2.6. Annexin V/PI Staining

**Figure 6:**
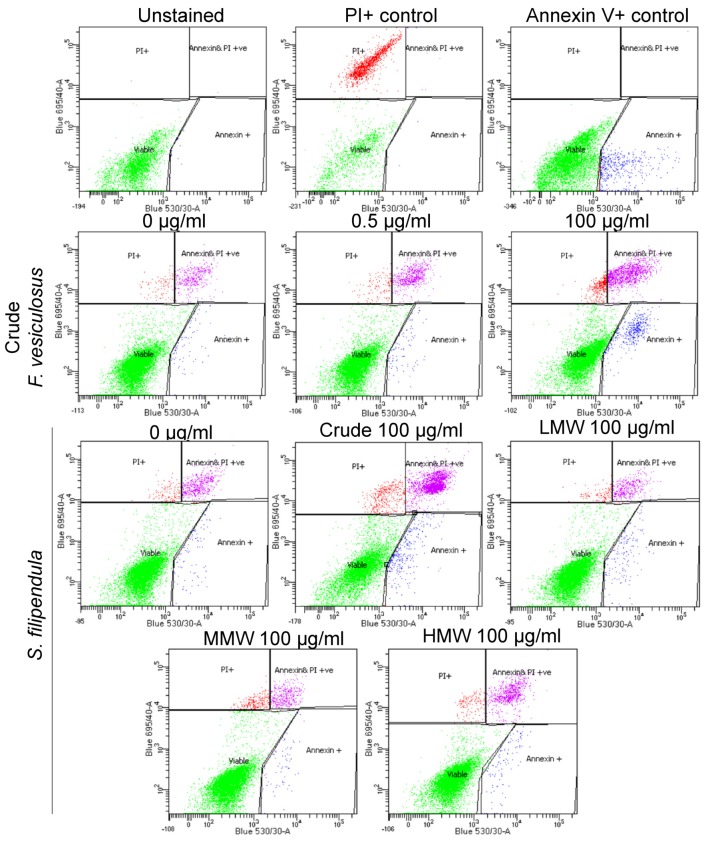
Annexin V/PI staining of MG63 cells under the effect of different fucoidans after 3 days of treatment. Representative population splits are shown. Nearly 10,000 single cell events were measured after staining live cells with Annexin V/PI and populations were split into viable (green events), early apoptotic (blue events), late apoptotic (pink events) and dead cells (red events).

To investigate if the cell death as a result of fucoidans treatment was due to apoptosis or necrosis, cells were seeded and treated with different fucoidans for three days. Crude fucoidan from *F. vesiculosus* at 100 μg/mL led to significantly fewer viable cells and more apoptotic or dead cells after treatment compared to respective vehicle control (0 μg/mL) (Figure 6, Supplementary Figure S4). This effect was dose-dependent. There were also reduced numbers of viable cells in samples treated with crude fucoidan from *S. filipendula* compared to the respective vehicle control (0 μg/mL) or other molecular weight fractions of fucoidan. Early apoptosis was more pronounced in crude fucoidan from *F. vesiculosus* and late apoptosis was more common in crude fucoidan from *S. filipendula* at the 100 μg/mL dose, indicative of differences in the mechanism of action of both fucoidans. Comparison of molecular weight fractions showed that there appeared to be more early and late apoptotic cells after treatment with 100 μg/mL HMW compared to 100 μg/mL LMW or MMW, though this effect was not statistically significant. There were more dead cells after treatment with MMW compared to LMW- and HMW-treated cells at the same dose. These results indicate that both MMW and HMW fractions were more toxic to cells and led to cell death, mostly via necrosis with some evidence of apoptosis.

### 2.7. Assessment of DNA Fragmentation in MG63 Cells after Fucoidan Treatment

**Figure 7:**
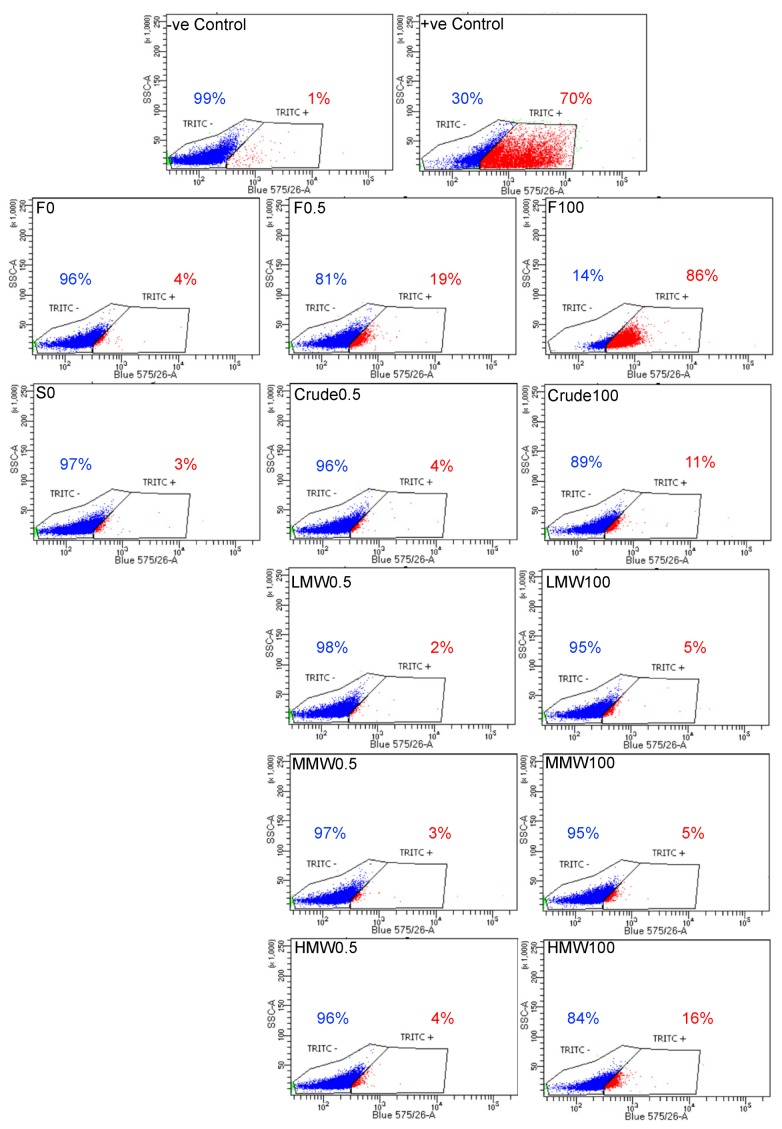
TUNEL assay for MG63 cells after 3 days treatment with different fucoidans. Nearly 6000 single cell events were measured, and populations were split into TRITC- (blue events, BrdU-) and TRITC+ (red events, BrdU+) using assay controls (*n* = 1). Notice the dose-dependent increase in TRITC+ cells after treatment with crude fucoidan from *F. vesiculosus* and HMW from *S. filipendula*.

Nuclear chromatin fragmentation is the hallmark of late-stage apoptosis, and in order to assess apoptosis-induced DNA fragmentation, a TUNEL assay was performed. There was a clear dose-dependent increase in cells positive for BrdU (TRITC-labelled) expression from 4% (for 0 μg/mL) to 86% (for 100 μg/mL) after treatment with crude fucoidan from *F. vesiculosus* (Figure 7). On the other hand, the increase was only from 3% (for 0 μg/mL) to 11% (for 100 μg/mL) after treatment with crude fucoidan from *S. filipendula*. Furthermore, when molecular weight fractions of fucoidan from *S. filipendula* were assessed, the results showed a very subtle increase in BrdU-expressing cells from 2%–3% (for 0.5 μg/mL) to 5% (for 100 μg/mL) after treatment with LMW and MMW. However, there was a four-fold increase after treatment with HMW from 4% (for 0.5 μg/mL) to 16% (for 100 μg/mL). These results clearly indicate that there can be different actions of fucoidan on chromatin material depending upon the species of algae as well as the molecular weight fraction of fucoidan used.

### 2.8. Effect of Fucoidan on Mitochondrial Health 

**Figure 8:**
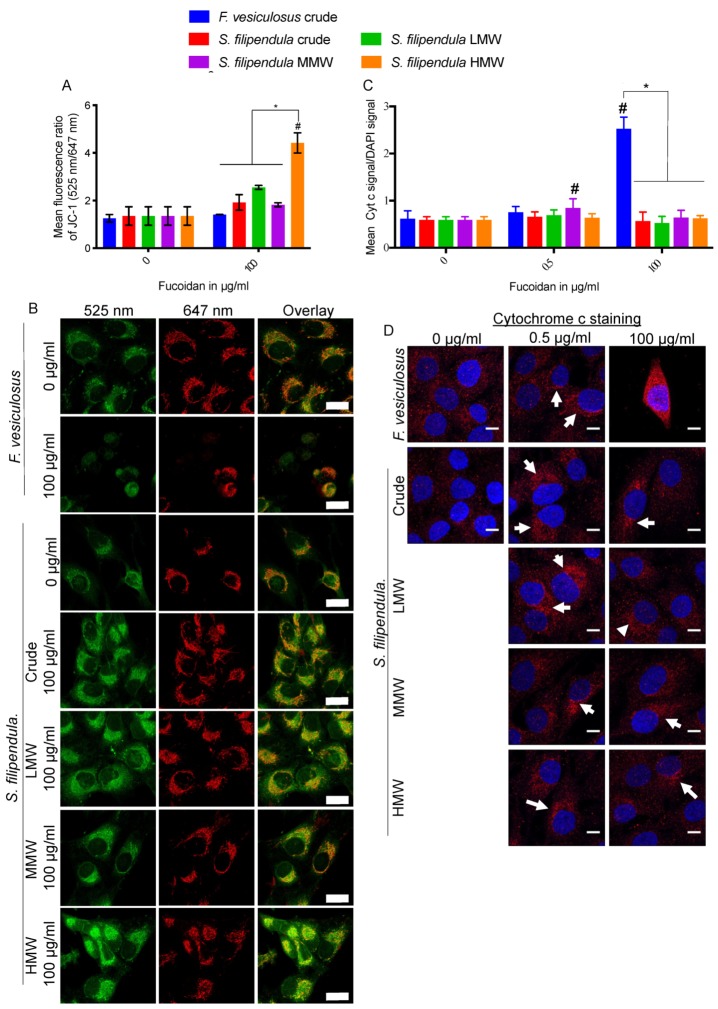
Assessment of mitochondria in MG63 cells after 3 days treatment with different fucoidans. (**A**) Mitochondrial membrane potential changes measured using JC-1 staining (at least 10 cells were analysed per condition). (**B**) Representative images of live cells taken under 525 nm (green) and 647 nm (red) channels with overlays. Scale bars—25 µm. (**C**) Mean ± SD of Cyt C signal to DAPI signal (*n* = 3 fields of view). (**D**) Representative overlay images of cells stained with Cyt C (Red) and DAPI (blue). Arrows—localization of Cyt c in perinuclear regions. Scale bar—10 µm. * *p* < 0.05, # *p* < 0.05 relative to respective vehicle controls.

To investigate if mitochondrial integrity was affected by fucoidan, live MG63 cells were stained with JC-1 after three days of fucoidan treatment. The results (Figure 8A,B) showed higher green-to-red ratios, and depolarization of mitochondrial membrane for all types of *S. filipendula* fucoidans at the 100 μg/mL dose, which was particularly high in the case of HMW. Interestingly, the lined features of J-aggregates (red channel) in cells treated with 100 μg/mL crude fucoidan from *F. vesiculosus* were reduced to small dots, indicative of mitochondrial fragmentation.

We further investigated the expression of Cyt C in MG63 after fucoidan treatment at the same time point (Figure 8C,D). There appeared to be the highest Cyt C expression across the cytoplasm of the entire cells after treatment with 100 μg/mL of crude fucoidan from *F. vesiculosus*. However, this increase was not seen in any of the other treated conditions and the expression was restricted to the perinuclear region. Collectively, the above results clearly indicate that fucoidan acts differently on mitochondria depending upon the species of algae and the molecular weight fraction.

### 2.9. Localization of Fucoidan in MG63 Cells

**Figure 9:**
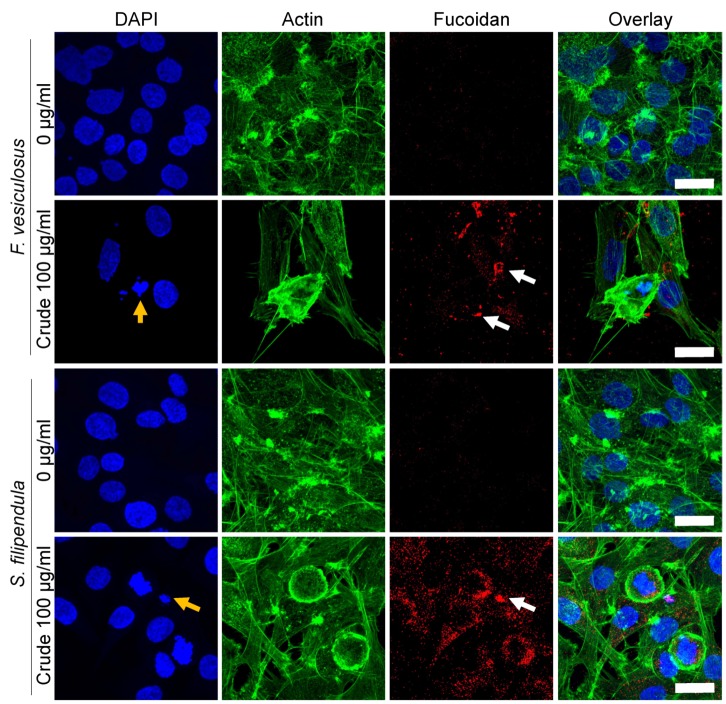
Penetration of different fucoidans in MG63 cells after 3 days of treatment. Max intensity z-projections for DAPI (blue)-, actin (green)- and fucoidan (red)-stained cells with overlay images are shown. At least 3 fields of view were analysed per condition. There are differences in patterns of staining for different crude fucoidan types (white arrows). Yellow arrows—condensed chromatin. Scale bars—25 µm.

To study the interaction of different fucoidans with MG63 cells, immunostaining for fucoidan was performed for cells treated for three days (Figure 9). There seemed to be a large amount of penetration of crude fucoidan from *S. filipendula* inside the cells, covering the entire cytosol. On the other hand, crude fucoidan from *F. vesiculosus* was only seen as clumps either near the edges of the cells or inside the cells, exhibiting limited penetration.

## 3. Discussion

Fucoidans from various different species of brown seaweed have been studied for anti-cancer applications. While most of the research revolves around more common forms of cancer, such as breast and colon cancer [6], use of fucoidan against osteosarcoma has recently been a keen area of interest [31,32,33], as even though it is a relatively less common cancer, the implications can be severe [34]. For the first time, here, we investigated the variable effects of crude fucoidan derived from two different species of brown algae, namely, *F. vesiculosus* (commercial source) and *S. filipendula* (derived from Colombian coast), and further evaluated the effect of different molecular weight fractions from 10–50 kDa (LMW), 50–100 kDa (MMW) and >100 kDa (HMW) fractions of fucoidan from *S. filipendula* on MG63 human osteosarcoma cells.

### 3.1. Fucoidan Causes G1 Phase Cell Cycle Arrest in MG63 Cells

A wide variety of previous studies reported cell cycle arrest in G1 or G2/M phase after fucoidan treatment [22,36,46]. A previous study showed that fucoidan from *F. vesiculosus* caused G0/G1 arrest of HT29 colon cancer cells after 24 h of treatment and it was dose-dependent (0, 50 and 100 μg/mL). This was associated with upregulation of p21WAF1 expression and suppression of cyclin D1, CDK 4, cyclin E and CDK 2 expression [46]. Similarly, another study also reported decreased expression levels of cyclin D1, cyclin E, CDK2 and CDK4 in bladder cancer cells in a dose-dependent manner (0, 25, 50, 100 μg/mL) after 24 h of fucoidan treatment (undefined species source) [47]. Atashrazm et al. [48] reported upregulated P21/WAF1/CIP1 expression and not cyclin D1 in APL cells treated for 24 h with *F. vesiculosus* fucoidan. Other fucoidans have also been reported to affect cell cycle in a similar way. Fucoidan from *U. pinnatifida* (commercially available or laboratory prepared) targeted p21Cip1/Waf and E2F-1 in PC-3 cells [38] and cyclin D1 and CDK4 in mouse hepatoma Hca-F cells [49]. In line with these studies, we showed that both crude fucoidans investigated led to cell cycle arrest in the G1 phase of cell cycle. However, their severity and mechanisms were different. *F. vesiculosus* fucoidan caused the cells to accumulate in the sub G1 phase of cell cycle, which is indicative of apoptotic population [50,51], while the *S. filipendula* fucoidan caused only a slight shift of population from the G2 phase to the G1 phase of cell cycle with no significant accumulation in the sub-G1 phase, and this seemed to be due to the MMW and HMW fractions of fucoidan within the crude extract.

### 3.2. F. vesiculosus Fucoidan Causes Stress-Induced Apoptosis-Like Cell Death in MG63 Cells 

Preliminary experiments for this study (Supplementary Figure S2) showed a slight increase in mitochondrial activity compared to control for doses below 100 μg/mL of *F. vesiculosus* fucoidan after one day of treatment, despite no significant difference in DNA content (used here as a measure of cell number), which strongly indicates that mitochondria may be more active in these cells than in control cells. This is in line with the observation that apoptosis is an active process and requires more ATP energy initially [52]. On Day three of culture however, JC-1 staining and TEM images confirmed that mitochondria in these cells were significantly reduced in number. The remaining mitochondria were dense, elongated and had a swollen cristae network, which may again indicate highly active mitochondria and accumulation of ions in the intermembrane space [53,54,55,56,57]. There seemed to be some mitochondria more swollen than the others and the cristae network seemed to be disintegrated within the mitochondria into a dense matrix, which suggested that the inner mitochondrial membrane may be intact. However, there may be damage to the outer mitochondrial membrane that eventually led to the release of Cyt C from the intermembrane space into the cytosol, thereby triggering the caspase cascade. This may have led to DNA fragmentation via endonuclease, seen via the TUNEL assay [58] and TEM (chromatin marginalization and condensation), and finally, apoptosis-like cell death, which was confirmed via Annexin V/PI staining. Some cells which may be more damaged may have also undergone necrosis, which would explain the presence of debris and some ruptured cells with damaged cell membranes or nuclear membranes [56,57]. Previously, similar mitochondrial damage and chromatin condensation was found to be associated with increased accumulation of high intracellular levels of ROS, depletion of glutathione, increased Bax-to-Bcl-2 ratio and activation of the caspase cascade in human hepatocellular carcinoma SMMC-7721 cells after *U. pinnatifida* treatment [59].

Indeed, several studies (reviewed in Reference [60]) claim that fucoidans, mainly from *F. vesiculosus* or *U. pinnatifida*, induce apoptosis via inhibition of phosphatidylinositol 3-kinase (PI3K)/Akt in human acute promyelocytic leukaemia (APL) cells [48] and PC-3 prostate cancer cells [38], via inhibition of the mitogen-activated protein kinase (MAPK) pathway in AML cells [48], MC3 human mucoepidermoid carcinoma cells [61] and DU-145 Prostate Cancer Cells [62], via downregulation of ID-1 (expressed in actively proliferating cells) in hepatocellular carcinoma cells [63], via downregulation of β-catenin in Eker rat leiomyoma tumour-derived cells [64] and PC-3 cells [38], and via generation of reactive oxygen species (ROS) in 5637 human bladder cancer cells [65], human hepatocellular carcinoma SMMC-7721 cells [59] and MCF-7 breast cancer cells [22,66]. Recently, commercial fucoidan from *U. pinnatifida* (average 130 kDa with 21% ± 3% fucose, 20% ± 5% galactose, 2% ± 2% mannose, and 30% ± 3% sulphate) was also shown to induce apoptosis in MG63 osteosarcoma cells characterised by increased roundness of the cells due to accumulation of F-actin at the cortex and increased expression of Annexin V together with chromatin condensation [33].

It is strongly argued that authentic apoptosis only occurs in the body and is irrelevant to non-renewable cell types, such as those used in vitro, as there is a lack of other cell types that play an important role in apoptosis [67]. Hence, stress-induced apoptosis-like cell death (SIaLCD) is often confused with apoptosis in most in vitro studies performed using cell lines [68,69,70]. Therefore, we postulate that MG63 cells underwent SIaLCD and not apoptosis in the present study. One of the targets of stress induction may have been in the ER, as TEM images also showed significant ER-like swelling and formation of vesicles inside the cytosol of MG63 cells that positively correlated to mitochondrial damage, all indicative of abnormal functioning of the ER and possible interplay between ER and mitochondria in these cells [69]. To alleviate this stress, autophagy can be triggered as a secondary response [71]. Recently, Hsu et al. [72] showed that both commercial fucoidans from *Laminaria japonica* and *F. vesiculosus* prevented lung cancer cells via ROS-mediated induction, Chen et al. [73] reported that commercial fucoidan from *F. vesiculosus* inhibited MDA-MB-231 breast cancer cells and HCT116 colon cancer cells by increasing the expression of CHOP (related to ER-stress), and Park et al. [74] reported that AGS human gastric adenocarcinoma cells had enhanced autophagy and mitochondrial damage after treatment with commercial fucoidan (undefined source).

### 3.3. S. filipendula Fucoidan Causes Necrosis in MG63 Cells

TUNEL assay showed that only 11% of cells treated with 100 µg/mL *S. filipendula* fucoidan were undergoing apoptosis-like death due to positive BrdU staining. On the other hand, the TEM images revealed condensation and marginalisation of chromatin material in the nuclei (in almost all cells, both intact or rupturing) complemented by damaged cell membranes, under the treatment of the same fucoidan at the same dose. This suggests that the chromatin changes were mostly random and that the mode of cell death in this case was most likely necrosis, oncosis or stress-induced necrosis-like cell death (SInLCD) and not only SIaLCD [56,69,75]. In support of this postulate was the presence of swollen mitochondria in these cells, wherein the cristae network was disorientated and marginalised towards the edges of the mitochondrial membrane, leading to the appearance of gaps inside the mitochondria. There were also some mitochondria that disintegrated within a dense matrix. All these signs are suggestive of accumulation of inner membrane components and possible interference with glycolysis, contributing to cell death. The fucoidan may also have affected other cell organelles such as ER, where it may have interfered with Ca^2+^ dynamics [76] and protein folding, thus causing build-up of misfolded or unfolded proteins inside ER. This stress may have again led to autophagy [77]. After irreversible damage in the cell however, fucoidan may have led to cell death [69].

In another study, Costa et al. [9] treated HeLa cells with crude fucoidan from *S. filipendula* for 24 h and found increased Annexin V expression, and the authors suggest that the cells underwent apoptosis via (glycogen synthase kinase-beta) GSK3β activation despite also reporting that there were no changes in caspase 9, caspase 3, p53, NFκB, ERK or p38 expression. Activated GSK-3β interacts with p53 to initiate the caspase cascade [78] and/or NFκB to activate the TNF signalling cascade [79,80] in order to induce apoptosis [81]. Hence, we further suggest that *S. filipendula* fucoidan may have also caused necrosis in those HeLa cells, similar as to that seen our present study.

We have shown, for the first time, variable localisation and penetration of two different types of brown seaweed crude fucoidans. Though the exact mechanism of cellular uptake is unclear, it is likely that structural variations in the two fucoidans may have led to variations in cell death mechanisms. Fucoidan from *F. vesiculosus* had twice as much sulphation as crude fucoidan from *S. filipendula*. This may have led to a globular structure of *F. vesiculosus* fucoidan causing its aggregation and clumping inside or around the cells and thus, exposing limited interaction sites to cellular components such as scavenger receptor (SR-B1, SR-BII, CD36) [82,83] and reduced uptake by the cells. This may have allowed for the cells to take their time and undergo SIaLCD [84]. On the other hand, the *S. filipendula* fucoidan may have a more linear molecular conformation, exposing relatively more reactive sites, which possibly also led to significantly more uptake of this fucoidan by the cells. As a consequence, it may have interfered with more than one molecular mechanism inside the cell or even led to irreparable DNA damage. Previously, extremely high ROS levels have been shown to cause necrotic cell death [58,85].

### 3.4. Fucoidan Affects Cell Cytoskeleton Formation and Adhesion

Cell adhesion to ECM is essential for invasion and proliferation of cancer cells and our results showed that all fucoidans from *F. vesiculosus* or *S. filipendula* inhibited MG63 cell attachment in a dose-dependent manner via inhibition of vinculin expression and accumulation of F-actin in the cell cortex (Figure 1 and Figure 9). However, this effect was more severe in the case of *F. vesiculosus* fucoidan. Previously, a similar mechanism of inhibition of attachment was observed for MDA-MB-231 adenocarcinoma cells after seeding in the presence of different doses of fucoidan from *Ascophyllum nodosum* [41] and MG63 cells treated with 100 μg/mL of commercial *U. pinnatifida* fucoidan [66]. In another study, fucoidan from *U. pinnatifida* was also found to downregulate l-selectin (an adhesion molecule) in Hca-F hepatocarcinoma cells [49]. 

### 3.5. HMW Fraction is Responsible for Cytotoxicity of Crude Fucoidan from S. filipendula

In this study, we consistently showed that greater inhibition of attachment, morphology, proliferation and extracellular matrix formation by MG63 cells occurred at higher molecular weights of *S. filipendula* fucoidan. These differences were clearly visible at lower doses and by 100 μg/mL, MMW and HMW fractions induced cytotoxicity at a similar level. The TUNEL assay also revealed an increase in dUTP-positive cells as the molecular weight of fucoidan increased. Furthermore, there was significantly more mitochondrial membrane depolarisation for HMW-treated cells compared to LMW- or MMW-treated cells. Interestingly, TEM images showed similar signs of toxicity as the crude fucoidan extract from *S. filipendula* and there was evidence that the nucleolus of the cells treated with HMW and MMW were fragmented, suggestive of these two fucoidans being more toxic. Altogether, these results strongly indicated that all three molecular weight fractions may have similar effects on MG63 cells to the crude fucoidan but fractions of >50 kDa were more severe than 10–50 kDa fractions. Moreover, as more than 93% of the crude fucoidan extract from *S. filipendula* had molecular weight >50 kDa, it seems that the MMW and HMW fractions defined the functionality of the crude extract.

Previously, the level and position of sulphation in fucoidan structure was considered to be an important factor that determines the bioactivity of fucoidan [86]. Cho et al. [20] studied the anti-cancer effects of 5–30 kDa and >30 kDa fractions of fucoidan from *U. pinnatifida* against the human stomach cancer cell line AGS and found that over-sulphation significantly enhanced the inhibition of cell growth for both molecular weight fractions. In this study, fucoidan from *F. vesiculosus* had the highest sulphation degree of nearly 34%, followed by MMW, HMW, crude extract and then LMW fractions of from *S. filipendula*. However, the degree of inhibition of MG63 attachment, viability, proliferation and ECM formation was not always in the same order. Hence, sulphation levels may not be the only determinant for the anti-cancer effect of fucoidans and there is likely to be a combinatorial effect of sulphation level, molecular weight and cell penetration ability of the fucoidans.

## 4. Materials and Methods

All reagents were purchased from Sigma-Aldrich (Gillingham, UK) unless otherwise stated.

### 4.1. Extraction and Purification of Fucoidan

Fucoidan from *F. vesiculosus* was sourced commercially (F5631, crude MWCO 20–200 kDa). *S. filipendula* was collected in October 2017 (for crude extract) and in January 2018 (for molecular weight fractions) in Santa Marta, Colombia (Otrosí number 5, Framework Contract for Access to Genetic Resources and their Derived Products N° 126 of 2016, register number RGE0156-5). The collected material was washed and dried at 50 °C in an oven for 6 h. The dried seaweed (DS) was coarsely powdered in a coffee grinder. The isolation of the fucoidan was carried out according to the methodology proposed by Synytsya et al. [87] with minor modifications. Sixty grams of the DS was added to 960 mL of HCl 0.1M and allowed to stand at 4 °C for 24 h. The resulting filtrate was neutralized with 1 M NaOH and precipitated with 3 volumes of ethanol. Then, the crude extract was obtained after centrifugation for 30 min at 4500 RPM. The resulting precipitate was re-dissolved in water and the pH was adjusted to 2 with 1 M HCl. An aliquot of 4 M CaCl_2_ was added to remove the precipitate and the supernatant was precipitated with 3 volumes of ethanol and re-dissolved in water. The enriched polysaccharide extract was further purified by dialysis (MWCO 12–14 kDa) for 48 h at 4 °C. The enriched crude extract (385 mg) obtained was fractionated by ultracentrifugation using MWCO filters of 10, 50 and 100 kDa and 3 molecular weight fractions were obtained: low (10–50 kDa, LMW, 24.0 mg), medium (50–100 kDa, MMW, 17.1 mg) and high (>100 kDa, HMW, 273.9 mg). Finally, the samples were lyophilized and stored in the dark. 

### 4.2. Measurement of Neutral Sugars in Fucoidan

Neutral sugars were measured using a phenol-sulfuric acid method [88]. Fifteen μL of each fraction were added to 400 μL of deionized water. The standards of fucose, glucose and the samples were mixed with 2 mL of sulfuric acid and mechanical stirring at 4000 RPM for 15 s. Subsequently, 400 μL of phenol (5%) was added and the resulting solution was heated at 90 °C for 5 min and cooled in a water bath. The absorbance was measured at 480 and 490 nm for fucose and glucose, respectively. The results were expressed based on the Equations (1) and (2) stated below:

(1) $$\%\;Neutral\;sugar\;\left(glucose\right)=\frac{A_{sample}-0.0536}{0.0309}*\;\frac{400}{V*10*W}$$

(2) $$\%\;Neutral\;sugar\;\left(fucose\right)=\frac{A_{sample}-0.0052}{0.0057}*\;\frac{400}{V*10*W}$$

where, *A_sample_* = absorbance of the sample at 490 nm, *V* = sample volume in μL and *W* = mass of the sample to obtain 1 mg/mL.

### 4.3. Measurement of Acid Sugars in Fucoidan

Acid sugars were measured using a carbazole-sulfuric acid method [89]. Forty μL of each fraction was added to 400 μL of deionized water. The samples and the standard d-glucuronic acid were mixed with 2 mL of sodium tetraborate (0.95 g/L sulfuric acid) and samples were heated at 100 °C for 12 min. Subsequently, 40 μL of carbazole (0.2% *w*/*v*, in ethanol) was added and heated at 100 °C for 10 min. The absorbance was measured at 525 nm. The results were expressed in terms of hexuronic acid based on Equation (3):

(3) $$\%\;Hexuronic\;acids=\frac{A_{sample}-0.1388}{0.0191}*\;\frac{1}{W}$$

where, *A_sample_* = absorbance of the sample at 525 nm and *W* = mass of the sample to obtain 1 mg/mL.

### 4.4. Measurement of Sulphated Sugars Content 

Fifty μL (1 mg/mL) of the fraction was mixed with 500 μL of deionized water. The samples and the standard of chondroitin sulphate were mixed with 4 mL of the DMB solution (containing 11 mg of 1,9DMB and 1 litre of sodium acetate 0.05 M, pH 4.75). The mixture was mechanically stirred at 4000 RPM for 15 s and left in the dark for 30 min. Absorbance was read at 520 nm [88,90]. The quantification of sulphated sugars was determinate using Equation (4):

(4) $$\%\;Sulphated\;sugars=\frac{A_{sample}-0.0275}{0.0065}*\;\frac{1}{W}$$

where, *A_sample_* = Absorbance of the sample at 520 nm and *W* = mass of the sample to obtain 1 mg/mL.

### 4.5. Culturing of MG63 Osteosarcoma Cells

MG63 osteosarcoma cells (passage 17 to 28) were maintained in α-MEM Eagle with sodium bicarbonate, 10% Foetal Bovine Serum (LabTech, Heathfield, UK), 2 mM l-Glutamine and 100 mg/mL Penicillin–Streptomycin at 37 °C and 5% CO_2_. At 90% confluence, cells were trypsinised, collected and seeded at 10,000 cells/cm^2^ on ibiTreat µ-Slide 8 well, 24-well plates, 6-well plates, T12.5 or T25 with working volumes of 250 µL, 500 µL, 2.5 mL, 2.5 mL and 5 mL, respectively. To assess cell attachment, cells were seeded in the presence of different fucoidans as described below, and after 24 h, assessments were performed. To assess proliferation or cell viability, treatments with different fucoidans were applied 24 h after cell seeding. 

### 4.6. Cell Metabolic Activity Assay

Metabolic activity was measured using PrestoBlue^®^ assay (Thermofisher Scientific, Hemel Hempstead, UK). At each time point, culture medium was removed and cells were washed with phosphate buffer saline (PBS) before adding PrestoBlue^®^ working solution (same volume as the culture medium) prepared by mixing PrestoBlue^®^ reagent with pre-warmed Hanks Balanced Salt Solution at a ratio of 1:9 (*v*/*v*). Three wells containing only PrestoBlue^®^ working solution with no cells were used as blanks. During the kinetic phase of reactions, 100 μL aliquots per well were taken after gentle mixing of cells and the fluorescence intensity was measured using 530 nm excitation and 590 nm emission filters on an Infinite F200 PRO TECAN fluorescence microplate (Tecan, Theale, UK). Cell metabolic activity was expressed after subtracting the reading for unreduced (blank) reagent and normalising to the control (0 μg/mL fucoidan dose) for respective fucoidan.

### 4.7. Actin Cytoskeleton and Focal Adhesion Staining

Culture medium was removed, and cells were washed once with PBS, before fixing them with 3.7% paraformaldehyde in PBS for 15 min at RT. Vinculin and actin staining was performed using the Actin Cytoskeleton and Focal Adhesion Staining Kit (Millipore, Watford, UK) as per the manufacturer’s instructions. Briefly, cells were washed twice with wash buffer (0.05% Tween-20 in Dulbecco’s Phosphate Buffered Saline (DPBS)) before permeabilizing with 0.1% Triton X-100 in DPBS for 5 min and then again washing with wash buffer. Cells were then blocked for 30 min using 1% Bovine serum albumin (BSA) in DPBS at RT, before adding anti-vinculin antibody (1:200 dilution) for 1 h at RT. Then, cells were washed twice using wash buffer before incubating in GTxMS, FITC conjugated secondary antibody (AP124F, Millipore, 1:100 dilution) and TRITC-labelled Phalloidin (P1951, Sigma, 1:1000 dilution) for 1 h at RT. Finally, cells were washed 2 times with wash buffer and counter stained with 10 μg/mL DAPI in DPBS for 5 min. Cells were visualised using a Nikon A1 Confocal microscope with a 60× oil lens, keeping the imaging parameters constant for quantification. For quantification of rounded cells, only actin and DAPI staining were performed and visualised using Eclipse Ti-E Nikon fluorescence microscope at 10×.

### 4.8. DNA Content Assay

The culture medium was removed, and the cells were washed with PBS before adding sterile deionised water (same volume as culture medium). Samples were lysed through three cycles of freeze thawing at −80 °C and then scraped using a sterile pipette tip. Cell lysates were diluted 1 in 10 by adding 10 μL lysates to 90 μL buffer provided by the kit. The test lysates were loaded on to a flat transparent 96-well plate (Grenier, Stonehouse, UK and DNA content was quantified using Quant-i™ PicoGreen^®^ dsDNA Assay kit (Thermofisher Scientific, Hemel Hempstead, UK) according to the manufacturer’s instructions. Briefly, 100 μL of PicoGreen working solution (1 in 200 dilution of dye with Tris-EDTA buffer) was added to each test well and three blank wells containing only water. After a 10 min incubation period at room temperature (RT), fluorescence intensity readings were taken on an Infinite F200 PRO TECAN fluorescence microplate using 480 nm excitation and 520 nm emission filters. The DNA concentration was extrapolated using a standard curve prepared from standard DNA calf thymus provided with the assay kit. DNA content was expressed after subtracting the reading of blank wells and normalising to the control (0 μg/mL fucoidan dose) for respective fucoidan.

### 4.9. Giemsa Staining

To assess cell morphology, cells were stained with Giemsa’s solution. It is a mixture of methylene blue, eosin and Azure B and stains the nucleus dark blue and cytoplasm blue to pink. Culture medium was removed, and cells were fixed with methanol for 10 s. Then, cells were washed with deionised water to remove any residual methanol before immersing them in Giemsa stain for 15 min at RT. Cells were then washed with deionised water to remove any residual stain and examined under a MOTIC^®^ AE 2000 inverted microscope at 40×.

### 4.10. Cell Cycle Analysis

Propidium iodide (PI) staining was performed to investigate cell cycle. Cells were seeded and left overnight to attach. The next day, culture medium was changed to alpha-MEM without serum for 16 h to synchronise their cell cycle and then cells were treated with fucoidan. On day 3 of treatment, medium was removed, and cells were washed twice with 1 mL of warm DPBS. After pelleting, 1 mL of 70% ethanol was added, and cells were vortexed immediately. Fixed cells were stored in a fridge until staining. For PI staining, fixed cells were washed twice with DPBS and then 300 µL of PI working solution (50 µg/mL PI in DPBS) and 50 µL of RNase working solution (0.3 µL/mL of RNase A in DPBS) was added. Cells were incubated at 37 °C for 1 h and then left overnight at 4 °C. The next day, samples were analysed using a BD LSRII flow cytometer and FlowJo software (BD, Wokingham, UK).

### 4.11. Annexin V/PI Staining

Annexin V/PI staining was performed to assess apoptosis or necrosis in cells. Cells were seeded and left overnight to attach. The next day, fucoidan treatment was started. On day 3 of treatment, Annexin V/PI staining was performed using the TACS^®^ Annexin V-FITC Kit (Trevigen, Gaithersburg, MD, USA). Briefly, the culture medium was collected in a tube, cells were washed with warm DPBS before trypsinising and collecting them in the same tube. Samples were pelleted using 300 g for 5 min, washed twice with DPBS and then stained with Annexin V and PI for 15 min at RT. The cells were again pelleted and washed with binding buffer provided with the kit before running the samples in an BD LSRII flow cytometer and analysing using FlowJo. For PI+ only and Annexin V+ only controls, cells were treated with 1% Saponin and 30% H_2_O_2_ solution respectively, for 10 min. To exclude autofluorescence signal, cells only (without any stain) were also included as a negative control group.

### 4.12. Transmission Electron Microscopy (TEM)

TEM was performed to examine the internal cell structure after fucoidan treatment. Cells were seeded and the next day, treatment was started. After 3 days, cells were collected by trypsinisation, washed with DPBS, pelleted and fixed in 2.5% glutaraldehyde/0.1 M PBS. Samples were subsequently treated with 2% buffered Tannic acid and 2% Osmium tetroxide, dehydrated in graded ethanol solutions, cleared in Epoxy Propane (EPP) and infiltrated in a 50/50 mix of EPP and araldite resin overnight on a lab rotor. Infiltration was completed with two changes of pure araldite resin before samples were embedded and polymerized for 72 hours at 60 °C. Ultrathin sections, approximately 85 nm thick, were cut on a Reichert Ultracet E ultramicrotome, picked up on copper EM grids and stained for 30 min with 3% aq. Uranyl Acetate, washed in water and stained with Reynold’s Lead Citrate for 5 min. Sections were examined on a FEI Tecnai Transmission Electron Microscope at an accelerating voltage of 80 Kv. Electron micrographs were recorded using an Orius 1000B Gatan digital camera and Digital Micrograph Software.

### 4.13. JC-1 Staining

The cyanine dye JC-1 (5,50,6,60-tetrachloro-1,10,3,30-tetraethylbenzimi- dazolylcarbocyanine iodide) (T3168, ThermoFisher Scientific, Hemel Hempstead, UK) enables discrimination of polarised and depolarised mitochondria. Normally JC-1 forms red fluorescent J-aggregates when concentrated in polarised mitochondria in response to their higher membrane potential. However, upon depolarisation of the mitochondrial membrane, JC-1 forms green monomers [91]. MG63 cells were seeded and left overnight to attach. The next day, treatment with fucoidan was started. On day 3 of treatment, culture medium was removed and cells were washed for 5 min with warm DPBS before incubating them in 10 µM of JC-1 dye in culture medium for 30 min. Dye was removed and cells were again washed with DPBS and visualised under ZEISS LSM 880 with Airyscan with a Plan-Apochromat 40×/1.3 oil lens using excitation at 488 nm and emission at 496–455 nm (for green monomers) and 576–719 nm (for red aggregates), keeping the imaging parameters constant for quantification.

### 4.14. Immunostaining for Cytochrome C (Cyt C) and Fucoidan

Culture medium was removed and cells were washed once with PBS, before fixing them with 3.7% paraformaldehyde in PBS for 10 min at RT. Samples were permeabilised with 0.1% Triton X-100 in DPBS for 1 h, then washed two times for 5 min with DPBS before blocking with 3% goat serum in 1% BSA/DPBS for 30 min. After removing the blocking solution, the samples were incubated overnight at 4 °C with BAM 3 (Hybridoma extracts containing anti-fucoidan primary antibody, 1:10 dilution) [92] or 1 µg/mL of anti-cyt c primary antibody (ab13575, Abcam, Cambridge, UK,) in blocking solution. The next day, samples were washed twice in DPBS for 5 min and then incubated with 1 µg/mL of Alexa Fluor 674 (red) goat anti-rat IgG preabsorbed (ab150167, Abcam) in blocking solution for 2 h at RT. Cells were again washed twice in DPBS for 5 min and counter stained with 100 nM of Acti-stain 488 phalloidin (PHDG1-A, Cytoskeleton, Inc., Denver, CO, USA) (if needed) and 10 μg/mL DAPI in 1% BSA/DPBS for 15 min at RT. Finally, cells were washed with DPBS and visualised using a Nikon A1 Confocal microscope with a 60× oil lens, keeping the imaging parameters constant for quantification. Cells with no primary or secondary antibody staining were used to exclude auto-fluorescence and controls with primary Ab only and secondary Ab only were also used to exclude non-specific Ab staining. Image analysis was performed using Image J Version: 2.0.0-rc-69/1.52p.

### 4.15. Terminal Deoxynucleotidyl Transferase dUTP Nick End Labelling (TUNEL) Assay

A TUNEL assay was performed to investigate DNA fragmentation in the latest stage of apoptosis. Cells were seeded and left overnight to attach. The next day, fucoidan treatment was started. On day 3 of treatment, a Guava TUNEL Assay kit (4500-0121, Millipore) was used to perform the assay. Culture medium was removed, and cells were washed with warm DPBS before trypsinising and collecting in sterile tubes. The cells were then pelleted by centrifuging at 300 g for 5 min and washed again with DPBS before fixing them with 3.7% paraformaldehyde in PBS for 20 min. Cells were stored at 4 °C in DPBS until staining. The TUNEL assay was performed as per the manufacturer’s instructions. Briefly, DPBS was removed from fixed cells, 70% ethanol (ice cold) was added and samples were stored in –20 °C for 2 h and then washed twice with wash buffer before adding the DNA labelling mix for 1 h at 37 °C. After this, rinsing buffer was added to the mix and cells were again pelleted. Supernatant was removed and cells were again incubated in anti-BrdU (TRITC-labelled) staining mix for 30 min at RT in the dark. Finally, rinsing buffer was added to the cells and analysed using a BD LSRII flow cytometer and FlowJo software. Positive and negative controls provided with the kit were used to select TRITC− and TRITC+ cells.

### 4.16. Statistical Analysis

All statistical analyses were performed using IBM SPSS Statistics 22. Mean and standard deviation were computed for at least three replicate samples in all experiments, except JC-1 stained images, where 2 fields of view were used and 10–15 cells per condition were assessed. For comparisons, one-way or two-way analysis of variance (ANOVA) was performed. Fucoidan type and concentration were two fixed factors. For pairwise comparisons, post-hoc analyses using Least Significant Difference (LSD), equivalent to no adjustments, were carried out. *P* values < 0.05 were considered significant. * indicates *p* < 0.05 for pairwise comparisons between groups shown, ^#^ indicates *p* < 0.05 relative to respective vehicle controls (0 µg/mL).

## 5. Conclusions

Our findings here showed that MG63 osteosarcoma cells are inhibited by fucoidans from two different brown algae, however the mechanism of action varies based on the penetrability of the fucoidan inside the cell. Moreover, as the molecular weight of fucoidan increases, its toxic effect also increases. Fucoidan from *S. filipendula* from the Colombian coast was, for the first time, investigated for a biomedical application, and this study provides evidence for its potential for anti-cancer therapy. 

## Figures and Tables

**Table 1:** Neutral sugars and uronic acid contents expressed as % of initial total mass and % of sulphation for the different fucoidans under study. Mean ± standard deviation (SD).

Fucoidan-Type	Neutral Sugars (%)		Uronic Acids (%)	Sulfation (%)
	Glucose	Fucose		
*F. vesiculosus* Crude	7.88 ± 0.04	56.57 ± 0.01	8.28 ± 0.01	33.92 ± 0.09
*S. filipendula* Crude	11.18 ± 0.01	79.48 ± 0.02	11.83 ± 0.01	16.52 ± 0.02
*S. filipendula* LMW	10.95 ± 0.01	76.69 ± 0.01	10.00 ± 0.04	15.05 ± 0.01
*S. filipendula* MMW	11.87 ± 0.01	82.31 ± 0.01	12.66 ± 0.01	25.99 ± 0.01
*S. filipendula* HMW	10.95 ± 0.02	70.28 ± 0.01	12.26 ± 0.01	20.13 ± 0.03

## Supplementary Materials

The following are available online at https://www.mdpi.com/1660-3397/18/2/104/s1, Figure S1: Assessment of MG63 cell morphology by actin staining, Figure S2: Toxicity of crude fucoidan derived from *F. vesiculosus* on MG63 cells after 1 day of treatment. Figure S3: Cell cycle analysis on Day 2 of treatment with 100 ug/mL of *S. filipendula* fucoidans (crude, LMW, MMW and HMW). Figure S4: Mean ± SD of viable, early apoptotic, late apoptotic and dead MG63 cells as seen through Annexin V/PI staining on day 3 of treatment with different fucoidans.
