# Supplementary material for: Fucoidan Inhibition of Osteosarcoma Cells is Species and Molecular Weight Dependent
Source: Mar Drugs. 2020 Feb 8;18(2):104. doi: 10.3390/md18020104 (PMC7074035; doi:10.3390/md18020104)
Supplement: Supplementary file 1 [file marinedrugs-18-00104-s001.pdf]

Supplementary

Fucoidan inhibition of osteosarcoma cells is species and molecular weight dependent

Dhanak Gupta[1,2], Melissa Silva[3], Karolina Radziun[1,2,4], Diana C. Martinez[1], Christopher Hill[5], Julie Marshall[2], Vanessa L. Hearnden[1], Miguel A. Puertas-Mejia[4] and Gwendolen C. Reilly[1,2*]

[1] Department of Materials Science and Engineering, University of Sheffield, UK.
[2] 2Insigneo Institute for in Silico Medicine, University of Sheffield, Sheffield, UK.
[3] Institute of Chemistry, University of Antioquia, Medellín, Colombia.
[4] Cell Bank, Department of Cell Biology, Faculty of Biochemistry, Biophysics and Biotechnology, Jagiellonian University, Krakow, Poland.
[5] Department of Biomedical Science, University of Sheffield, Sheffield, UK.

*Correspondence: Department of Materials Science and Engineering and INSIGNEO Institute for in silico Medicine, The Pam Liversidge Building (C+04), The Sir Robert Hadfield Building, Mappin Street, Sheffield, S1 3JD, UK. E-mail: g.reilly@sheffield.ac.uk

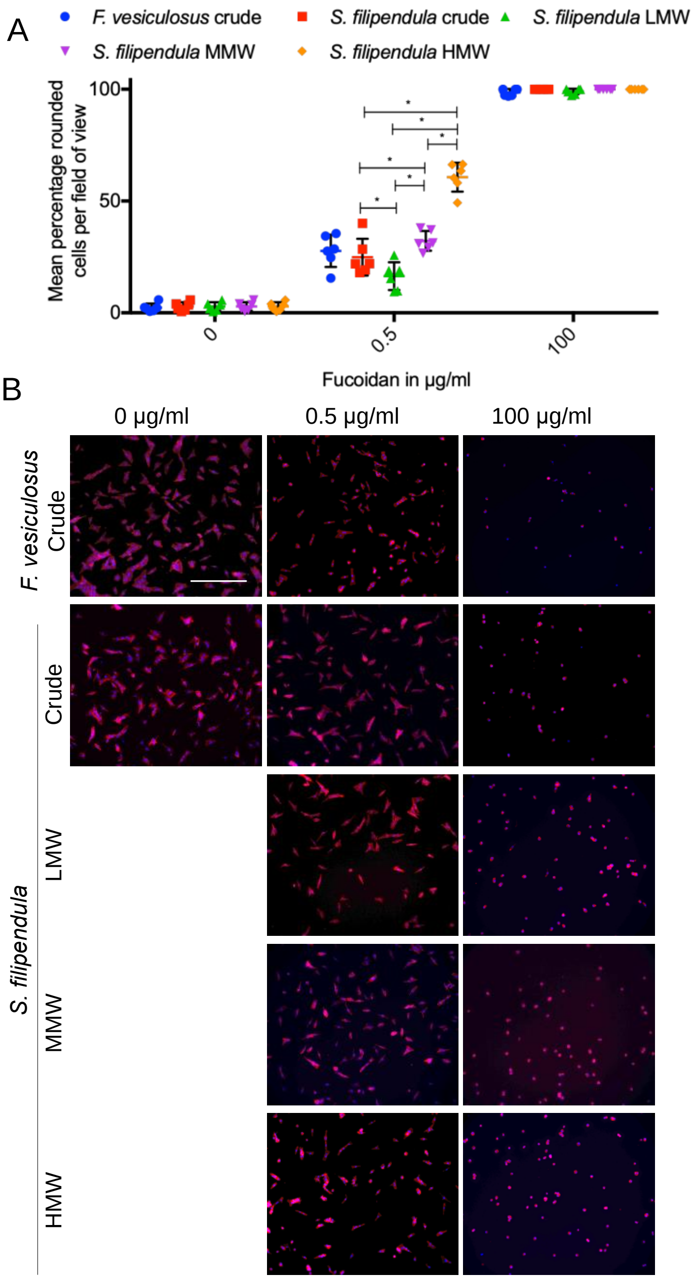

Figure S1: Assessment of MG63 cell morphology by actin staining. A) Mean ± S.D. of MG63 cells with round morphology after seeding in the presence of different doses of fucoidans (n = 6). B) Representative overlay images of actin (red) and DAPI (blue) stained MG63 cells treated with 0, 0.5 and 100 µg/ml of different fucoidan. Scale bar, 500 µm. * $p < 0.05$, # $p < 0.05$ relative to respective vehicle controls.

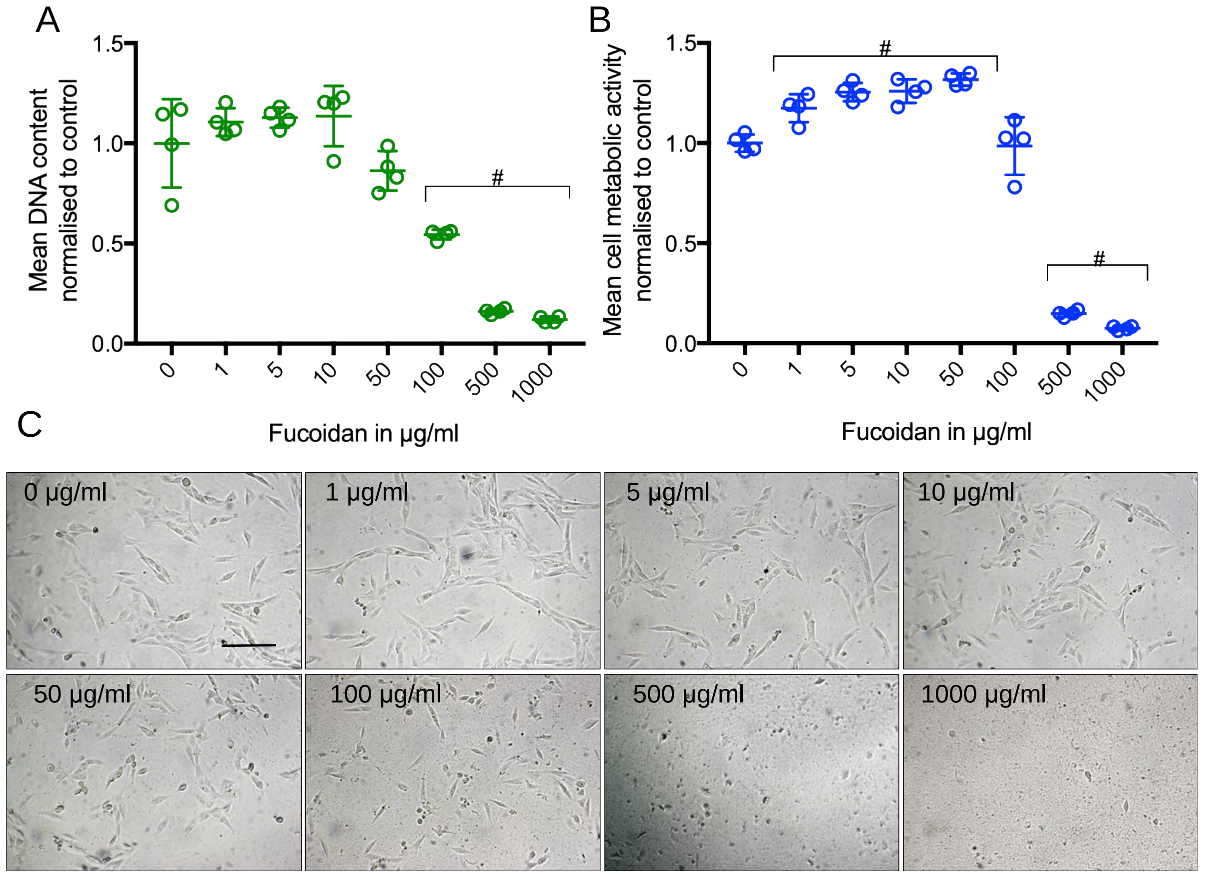

Figure S2: Toxicity of crude fucoidan derived from *F. vesiculosus* on MG63 cells after 1 day of treatment. Presto Blue™ assay for cell metabolic activity and Pico Green assay for DNA content measurements were performed (n = 4). Scale bar, 200 µm. Mean ± S.D. values are shown. # $p < 0.05$ relative to vehicle control.

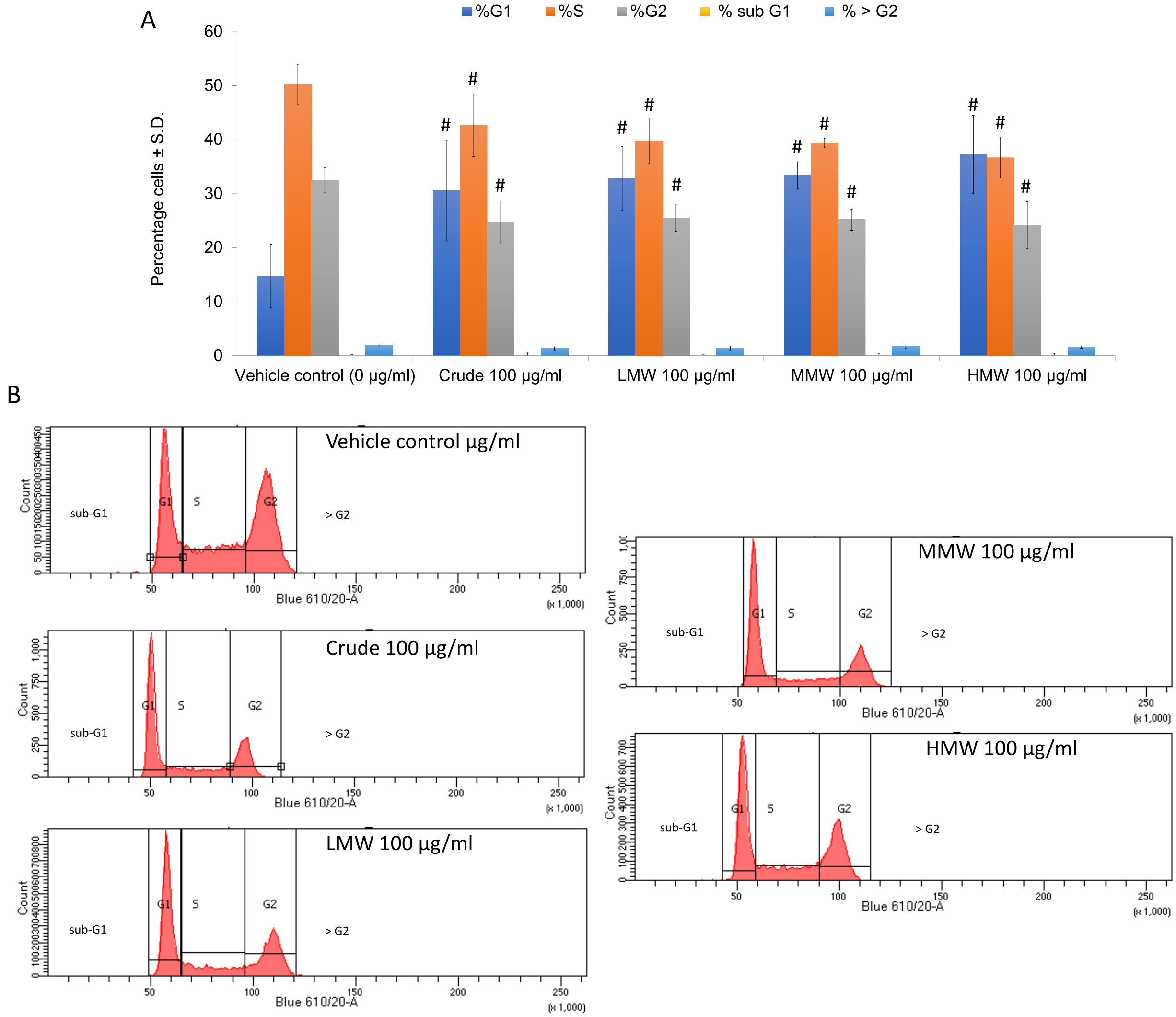

Figure S3: Cell cycle analysis on Day 2 of treatment with 100 ug/ml of *S. filipendula* fucoidans (crude, LMW, MMW and HMW). (A) Mean and S.D. of cells in different phases of cell cycle (n = 3). (B) Representative histograms of cells in sub-G1, G1, S, G2 and > G2 phases in different conditions. There was consistent rise in G1 populations and drop in S and G2 populations after treatment with any of the fucoidans compared to vehicle control (0 ug/ml) and this clearly shows G1 arrest due to these fucoidans. # $p < 0.05$ relative to respective vehicle controls.

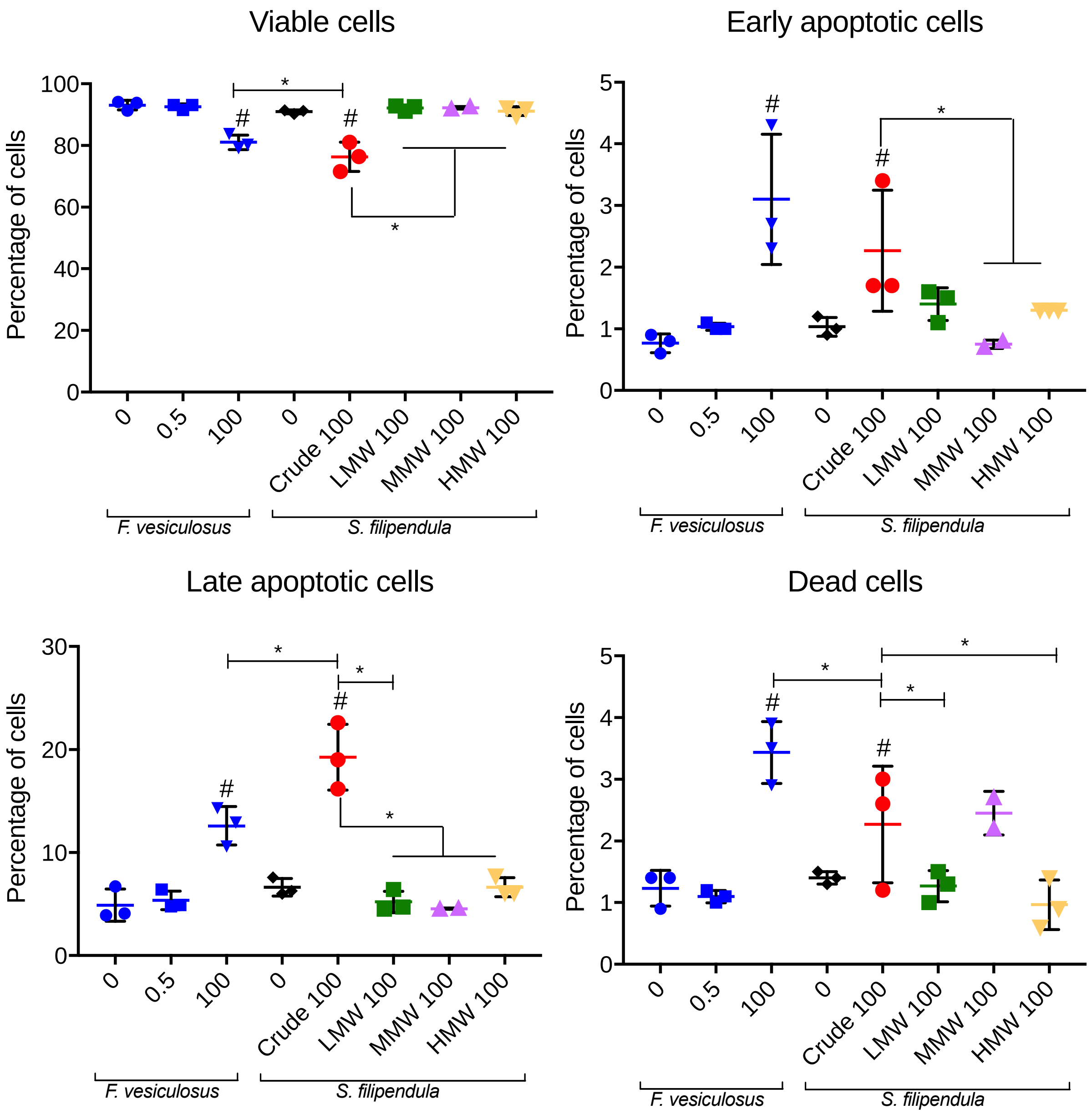

Figure S4: Mean ± S.D. of viable, early apoptotic, late apoptotic and dead MG63 cells as seen through Annexin V/PI staining on day 3 of treatment with different fucoidans. * $p < 0.05$, # $p < 0.05$ relative to respective vehicle controls.
